# SgNramp1, a plasma membrane-localized transporter, involves in manganese uptake in *Stylosanthes guianensis*

**DOI:** 10.3389/fpls.2022.1027551

**Published:** 2022-10-06

**Authors:** Xiaoyan Zou, Rui Huang, Linjie Wang, Guihua Wang, Ye Miao, Idupulapati Rao, Guodao Liu, Zhijian Chen

**Affiliations:** ^1^ Key Laboratory of Tropical Crops Germplasm Resources Genetic Improvement and Innovation of Hainan Province, Institute of Tropical Crop Genetic Resources, Chinese Academy of Tropical Agricultural Sciences, Haikou, China; ^2^ College of Tropical Crops, Hainan University, Haikou, China; ^3^ Rubber Research Institute, Chinese Academy of Tropical Agricultural Sciences, Haikou, China; ^4^ Crops for Nutrition and Health, Alliance of Bioversity International and International Center for Tropical Agriculture, Cali, Colombia

**Keywords:** nramp, trace metal, metal homeostasis, gene expression, subcellular localization

## Abstract

Transporters belonging to the natural resistance-associated macrophage protein (Nramp) family play important roles in metal uptake and homeostasis. Although Nramp members have been functionally characterized in plants, the role of Nramp in the important tropical forage legume *Stylosanthes guianensis* (stylo) is largely unknown. This study aimed to determine the responses of *Nramp* genes to metal stresses and investigate its metal transport activity in stylo. Five *SgNramp* genes were identified from stylo. Expression analysis showed that *SgNramp* genes exhibited tissue preferential expressions and diverse responses to metal stresses, especially for manganese (Mn), suggesting the involvement of *SgNramps* in the response of stylo to metal stresses. Of the five *SgNramps*, *SgNramp1* displayed the highest expression in stylo roots. A close correlation between *SgNramp1* expression and root Mn concentration was observed among nine stylo cultivars under Mn limited condition. The higher expression of *SgNramp1* was correlated with a high Mn uptake in stylo. Subsequent subcellular localization analysis showed that SgNramp1 was localized to the plasma membrane. Furthermore, heterologous expression of *SgNramp1* complemented the phenotype of the Mn uptake-defective yeast (*Saccharomyces cerevisiae*) mutant Δ*smf1*. Mn concentration in the yeast cells expressing *SgNramp1* was higher than that of the empty vector control, suggesting the transport activity of SgNramp1 for Mn in yeast. Taken together, this study reveals that SgNramp1 is a plasma membrane–localized transporter responsible for Mn uptake in stylo.

## Introduction

Trace metals, such as manganese (Mn), iron (Fe), zinc (Zn), and copper (Cu), are essential elements for plant growth and productivity. These metal ions involve in multiple physiological and biochemical processes in plants, such as stabilizing the structure of many biomacromolecules and serve as cofactors for key proteins and enzymes (Marschner, 2012). For example, Mn is an essential component in the Mn cluster of the oxygen-evolving complex in photosystem II (PSII) that is required for the electron transport chain in photosynthesis. Mn also acts as a critical cofactor of numerous enzymes, such as superoxide dismutase (SOD), catalase (CAT), decarboxylases, and RNA polymerases (Chen and Li, 2021). Despite its necessity at a low dose, the metal ions presented at excess levels can be harmful to plants, which are similar to the toxic effects of non-essential heavy metals, such as aluminum (Al), cadmium (Cd), arsenic (As), and lead (Pb). The excess amount of trace elements and heavy metals can cause phytotoxicity to plant cells, such as triggering oxidative stress, inhibiting enzyme activity, impeding the structure and function of photosynthetic apparatus, and damaging respiration and energy metabolism, ultimately inhibiting plant growth (Fryzova et al., 2018; Huang et al., 2020; Riyazuddin et al., 2021).

To maintain normal growth, plants have to regulate metal uptake and homeostasis in response to the fluctuated metal status in soils from deficient to toxic levels, which can be achieved *via* a variety of metal transporters, such as members of the natural resistance-associated macrophage protein (Nramp), Zn-regulated transporter/Fe-regulated transporter-like protein (ZRT/IRT), metal tolerance protein (MTP), and heavy metal ATPase (HMA) (Wang et al., 2020; Kaur et al., 2021; Pottier et al., 2022). Among these metal transporters, Nramp, an integral membrane protein, is a critical transporter for divalent metals. Most Nramp proteins include a unique motif and 10–12 conserved transmembrane domains (TMDs) (Li et al., 2021b). Nramp proteins have been characterized to transport a wide range of metal ions across the cellular membrane, including Mn, Fe, Zn, Cd, or Al (Li et al., 2019). To date, a set of Nramp members have been identified in various plants, such as Arabidopsis (Thomine et al., 2000; Li et al., 2022), rice (*Oryza sativa*) (Xia et al., 2010; Sasaki et al., 2012; Mani and Sankaranarayanan, 2018; Chang et al., 2020), *Medicago truncatula* (Tejada-Jiménez et al., 2015), soybean (*Glycine max*) (Kaiser et al., 2003; Qin et al., 2017), barley (*Hordeum vulgare*) (Wu et al., 2016) and tobacco (*Nicotiana tabacum*) (Liu et al., 2022).

For example, six Nramp members are identified in Arabidopsis, and five of them have been functionally characterized (Gao et al., 2018; Li et al., 2022). Among them, AtNramp1 is mainly expressed in roots and is a plasma membrane (PM)–localized transporter responsible for the high-affinity Mn uptake in Arabidopsis (Cailliatte et al., 2010). In addition to Mn, AtNramp1 can also transport Fe and cobalt (Co) *in vivo* (Curie et al., 2000; Cailliatte et al., 2010). AtNramp2 is found to be localized to the trans-Golgi network and is implicated in remobilization of Mn in Golgi, which is required for Arabidopsis root growth under Mn deficiency (Gao et al., 2018). It has been demonstrated that both AtNramp3 and AtNramp4 are induced by Fe deficiency and are essential for vacuolar Fe mobilization during seed germination in Arabidopsis (Thomine et al., 2003; Lanquar et al., 2005). Both AtNramp3 and AtNramp4 can rescue the growth of the yeast (*Saccharomyces cerevisiae*) mutant Δ*smf1*, which is defective in Mn uptake (Thomine et al., 2000); mutation analysis reveals the roles of AtNramp3 and AtNramp4 in intracellular Mn homeostasis in Arabidopsis (Lanquar et al., 2010). AtNramp6 can transport Fe and Cd in yeast cells, and disruption of AtNramp6 reduces lateral root growth in Arabidopsis under Fe deficiency (Cailliatte et al., 2009; Li et al., 2019). Recently, it has been reported that AtNramp6 together with AtNramp1 is cooperatively involved in Mn utilization in Arabidopsis (Li et al., 2022). The transport activities of Nramp homologues for various metal ions are also demonstrated in other plant species, including OsNramps from rice for Mn, Fe, Cd and Al (Xia et al., 2010; Yamaji et al., 2013; Peris-Peris et al., 2017; Li et al., 2021c), MtNramp1 from *M. truncatula* for Fe and Mn (Tejada-Jiménez et al., 2015), HvNramp5 from barley for Mn and Cd (Wu et al., 2016), MhNramp1 from apple (*Malus hupehensis*) for Cd (Zhang et al., 2020), FeNramp5 from buckwheat (*Fagopyrum esculentum*) for Mn and Cd (Yokosho et al., 2021), and NtNramp1 from tobacco for Fe and Cd (Liu et al., 2022).

Although the functions of Nramp homologues have been characterized and analyzed in different plants, the role of the Nramp members in metal transport activity remains to be elucidated in the tropical forage legume, *Stylosanthes guianensis* (stylo). Stylo is an acid soil adapted, pioneer forage legume, which is widely used for multipurpose including land reclamation and restoration, soil quality improvement, and fodder for farm animals (Tang et al., 2009; Marques et al., 2018; Guo et al., 2019). Stylo displays superior level of tolerance to nutrient and metal stresses that are common in acid soils of the tropics, including phosphorus (P) deficiency, excess Mn stress, and Al toxicity (Sun et al., 2014; Chen et al., 2015; Chen et al., 2021). Considering the key role of Nramp in metal ion uptake and homeostasis, this study aimed to determine the responses of *Nramp* genes to metal stress and investigate its roles in metal transport activity. Five *SgNramps* were identified from stylo and their expression patterns were analyzed. A PM-localized transporter SgNramp1 was further demonstrated to be involved in Mn uptake. The results of this study provide a candidate gene for breeding stylo cultivars with high-efficient Mn uptake.

## Materials and methods

### Identification of *nramp* genes in *S. guianensis*

Five *Nramp* genes were isolated from the previously reported transcriptomic data in stylo (Jiang et al., 2018; Jia et al., 2020; Chen et al., 2021). The full length of each *Nramp* was amplified from the cDNA library stock of stylo roots (Song et al., 2022). These five *Nramp* genes were named from *SgNramp1* to *SgNramp5* under GenBank accession numbers ON338040, ON338041, ON338042, ON338043, and ON338044, respectively. ClustalX and MAGA4 were used to perform multiple alignment and phylogenetic analyses, respectively. TMD was predicted by the HMMER (https://www.ebi.ac.uk/Tools/hmmer/search/hmmscan). The conserved motifs of Nramp proteins were analyzed by MEME (https://meme-suite.org/meme/tools/meme) and Pfam (http://pfam.xfam.org/search/sequence) programs with default parameters.

### Plant growth and treatments

In this study, the stylo cultivar “Reyan NO.5” was used for gene expression analysis. Stylo seeds were sown on moistened filter papers at 25°C in the dark for 2–3 days. The germinated seedlings were then transplanted into Hoagland solution as previously described (Song et al., 2022). The macronutrients and micronutrients in the modified Hoagland solution included 2 mM Ca(NO_3_)_2_, 3 mM KNO_3_, 0.25 mM KH_2_PO_4_, 0.5 mM MgSO_4_, 25 μM MgCl_2_, 80 μM Fe-Na-EDTA, 5 μM MnSO_4_, 0.5 μM ZnSO_4_, 1.5 μM CuSO_4_, 0.09 μM (NH_4_)_6_Mo_7_O_24_, and 23 μM Na_2_B_4_O_7_. The pH of the solution was adjusted to 5.8 every 2 days. Stylo seedlings were grown in a greenhouse at 25–32°C under normal sunlight conditions with a photoperiod of about 13h light. For expression analysis of *SgNramps*, root, stem, and leaf tissues were harvested at 21 days of growth, whereas flower and seed were harvested at 120 days and 140 days, respectively.

To investigate the responses of *SgNramps* to deficiencies of Mn, Fe, Zn, and Cu, the 14-day-old stylo seedlings precultured in half-strength Hoagland solution were transferred to nutrient solution without Mn, Fe, Zn, or Cu application for 7 days. Leaf and root were harvested and used to gene expression analysis. To analyze the effects of excess Mn, Fe, Zn, and Cu treatments on the transcripts of *SgNramps*, the 14-day-old stylo seedlings precultured in half-strength Hoagland solution were transplanted into fresh Hoagland solution supplied with 400 μM MnSO_4_, 800 μM Fe-Na-EDTA, 20 μM ZnSO_4_, or 10 μM CuSO_4_ for excess Mn, Fe, Zn, or Cu treatments, respectively. After 7 days of treatments, leaf and root were harvested and used for gene expression analysis. The stylo seedlings grown in full-strength Hoagland solution for 21 days were set as the control treatment.

In addition, nine stylo cultivars belonging to *S. guianensis*, including “Reyan NO.2”, “Reyan NO.5”, “Reyan NO.10”, “Reyan NO.13”, “Reyan NO.18,” “Reyan NO.20”, “Reyan NO.21”, “Reyan NO.22”, and “Reyan NO.24”, were used to evaluate the genotypic variations in *SgNramp1* expression. Fourteen-day-old stylo seedlings precultured in half-strength Hoagland solution as described above were transplanted into fresh Hoagland solution supplied with 0.1 or 5 μM MnSO_4_ regarding as low Mn and normal Mn treatments, respectively. After 7 days of Mn treatments, roots were harvested for *SgNramp1* expression and Mn concentration analyses.

### Quantitative real-time polymerase chain reaction analysis

Total RNA was extracted using TRIzol reagent (TIANGEN Biotech, China), and then it was used to synthesize first strand cDNA *via* the HiScript III cDNA synthesis kit (Vazyme, Nanjing, China). The synthesized cDNA was further used to perform quantitative real-time polymerase chain reaction (qRT-PCR) analysis using SYBR Green Master mix reagent (Vazyme, Nanjing, China). qRT-PCR reaction was detected by a QuantStudio™ 6 Flex Real-Time equipment (Thermo Fisher Scientific, Massachusetts, USA). Gene specific primers for qRT-PCR analysis are detailed in **Supplementary Table S1**. The expression of each *SgNramp* was calculated relative to the housekeeping gene *SgEF-1a* (Song et al., 2022). Three biological replicates were included in this experiment.

### Analysis of Mn concentration

After samples thoroughly dried in an oven for 7 days, the dried samples were digested with concentrated nitric acid at 140°C. Mn concentration in the digested solution was determined by atomic absorption spectroscopy. The standard reference material (GBW07603) was used to validate Mn determination as previously described (Li et al., 2021a).

### Subcellular localization of SgNramp1

The open reading frame (ORF) of *SgNramp1* was amplified by PCR using *SgNramp1-GFP* primers (**Supplementary Table S1**). The PCR product was cloned into the N-terminus of the green fluorescent protein (GFP) of the *pBWA(V)HS* vector (Li et al., 2021a). The constructs were introduced into *Agrobacterium tumefaciens* strain Gv3101 and were then transiently expressed in leaves of 5-week-old tobacco (*Nicotiana benthamiana*) seedlings according to Li et al. (2021). The PM marker (OsMCA1) (Kurusu et al., 2012) fused with red fluorescence protein (mKATE) was used to co-localization with SgNramp1 or empty vector. The fluorescence was detected by a confocal laser scanning TCS SP8 microscopy (Leica, Wetzlar, Germany). GFP fluorescence was detected at 500–530 nm, whereas the red fluorescence was detected at 580–630 nm.

### Metal transport analysis of SgNramp1 in yeast cells

In this study, the Mn uptake-defective mutant Δ*smf1* (MATa; his3Δ1; leu2Δ0; met15Δ0; ura3Δ0; YOL122c::kanMX4), the Fe uptake-defective mutant Δ*fet3fet4* (MATa; his3Δ1; leu2Δ0; met15Δ0; ura3Δ0; YMR058w::kanMX4) and the Cd-sensitive mutant Δ*ycf1* (MATa; his3Δ1; leu2Δ0; met15Δ0; ura3Δ0; YDR135c::kanMX4) were obtained from the Euroscarf. The ORF of *SgNramp1* was amplified using *SgNramp1-pYES2* primers (**Supplementary Table S1**). The amplified product was cloned into the yeast expression vector *pYES2* (Invitrogen, Carlsbad, United States). The construct and empty vector were then introduced into the above yeast mutants using the LiOAc/PEG method. The yeast transformants were incubated in a liquid synthetic complete (SC–U/Glu) medium consisting of yeast nitrogen base, glucose, and amino acids without uracil at 30°C for about 6 h. After optical density (OD_600_) of the yeast cells reached to 0.6, the yeast cells were diluted to an OD_600_ of 0.2 and three 10-fold serial dilutions were prepared. Then, 10 μl of each dilution was spotted onto the induction SC–U plates containing 2% (w) galactose (Gal) and 1% raffinose according to Li et al. (2021a). For Mn transport analysis in Δ*smf1*, the plate was added with 0, 20, and 25 mM Mn-chelator ethylene glycol tetraacetic acid (EGTA). For Fe transport analysis in Δ*fet3fet4*, the plate was added with 0, 20, and 40 µM FeSO_4_. For assay on the tolerance to Cd in Δ*ycf1*, the plate was added with 0, 40, and 60 µM CdCl_2_. The yeast cells were incubated at 30°C for 2 d prior to photographing.

To assess yeast growth in liquid medium, the transformed yeast strain Δ*smf1* was precultured in SC–U liquid medium containing 2% (w) Gal and 1% raffinose to the log phase. The precultured yeast cells were diluted to an OD_600_ of 0.05 and were then grown in SC-U liquid medium added with or without 20 mM EGTA. The OD_600_ value of yeast cells was measured from 0-48h. To determine Mn uptake in yeast, the yeast cells with an initial OD_600_ of 0.2 were incubated in SC–U liquid medium supplemented with 20 and 40 μМ MnSO_4_ for 24 h. To analyze kinetics of Mn uptake, the yeast cells with an initial OD_600_ of 0.2 were incubated in SC–U liquid medium supplemented with 0–10 μМ MnSO_4_ for 6 h. After treatments, the yeast cells were harvested and washed with 5 mM CaCl_2_ solution, followed by washing with deionized water twice. The collected cells were used for Mn determination. The *K*_m_ and *V*_max_ values of Mn uptake in yeast cells expressing *SgNramp1* were estimated by Lineweaver-Burke plots of Mn concentrations at various Mn treatments after subtracting the Mn concentration in yeast cells transformed with *pYES2* empty vector.

### Statistical analyses

One-way ANOVA and Student’s *t*-test were analyzed by using SPSS13.0 (SPSS Institute, Chicago, United States).

## Results

### Characterization of *SgNramp* genes in *S. guianensis*

**Figure 1:**
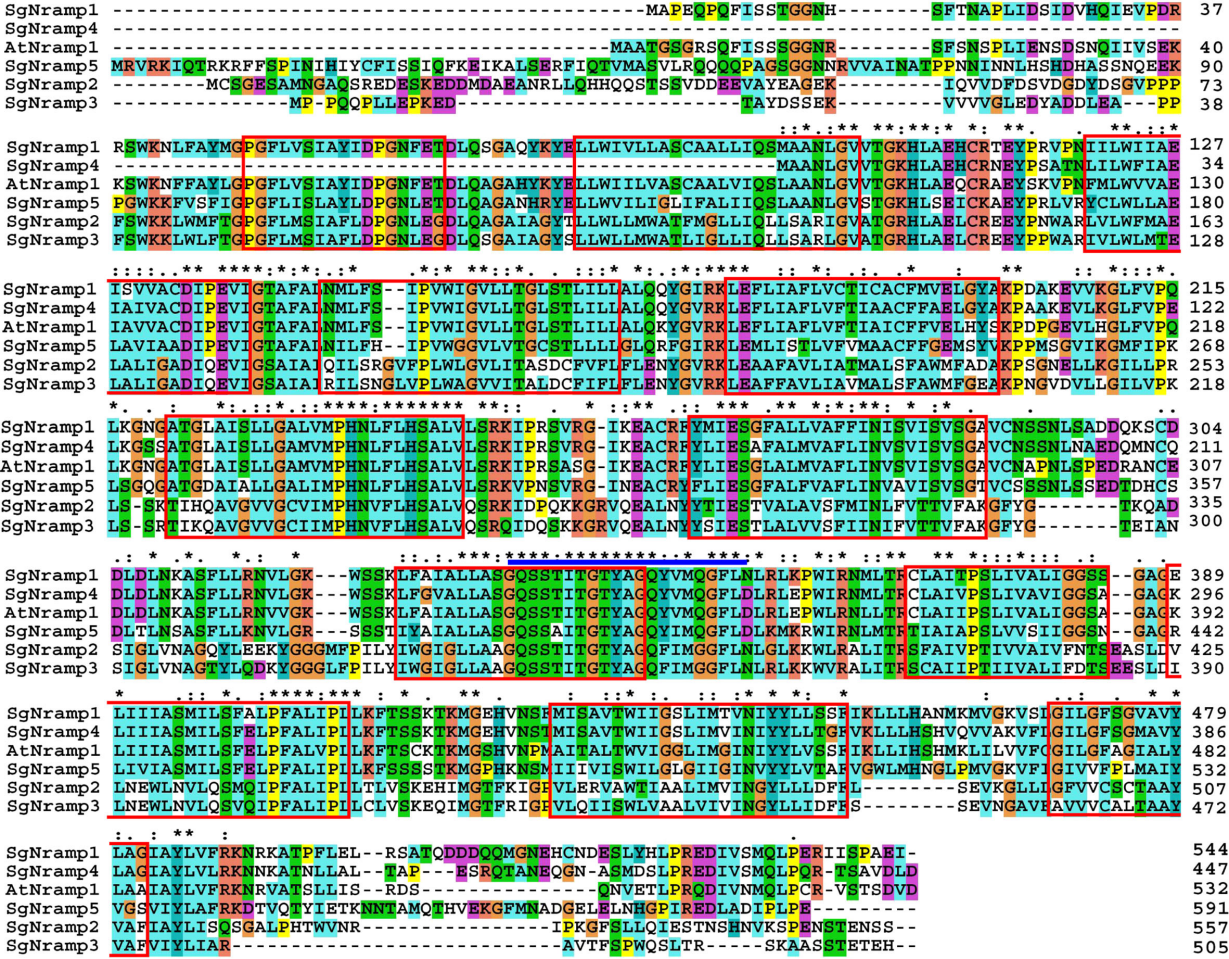
Multiple alignment of SgNramps from *S. guianensis* and AtNramp1 from Arabidopsis. The transmembrane domains (TMDs) were boxed with red color. The unique amino acid residues of the Nramp proteins were labeled with blue line above the sequence. The following Nramp proteins were used: SgNramp1 (ON338040), SgNramp2 (ON338041), SgNramp3 (ON338042), SgNramp4 (ON338043), SgNramp5 (ON338044), and AtNramp1 (At1g80830). * in the figure indicates the same amino acid residue included in all Nramp proteins.

In this study, five *Nramp* genes were cloned in stylo, which were named as *SgNramp1* to *SgNramp5* (**Supplementary Table S2**). The full-length sequences of *SgNramps* varied from 1,344 to 1,779 bp, and they encoded proteins differing from 447 to 591 amino acid residues in length with molecular weight ranging from 48.3 to 64.5 kDa (**Supplementary Table S2**). All of the SgNramp members possessed the conserved Nramp domain (PF01566). Among them, four SgNramp (SgNramp1, 2, 3, and 5) proteins included 12 conserved TMDs, whereas SgNramp4 harbored 10 TMDs (**Figure 1** and **Supplementary Table S2**). Furthermore, the SgNramp proteins carried the unique amino acid residues GQSST(/A)ITGTYAGQF(/Y)I(/V)MQ(/G)GFLN(/D) of plant Nramp proteins (**Figure 1**). The amino acid sequences of five SgNramps shared 31.8–81.2% identities with each other (**Supplementary Figure S1**). In addition, SgNramp1 to SgNramp5 shared 32.1–77.0% homology identities with AtNramp1 in Arabidopsis, 35.9–76.0% identities with MtNramp1 in *M. truncatula* and 34.1–81.8% homology identities with GmDMT1 in soybean (**Supplementary Figure S1**).

**Figure 2:**
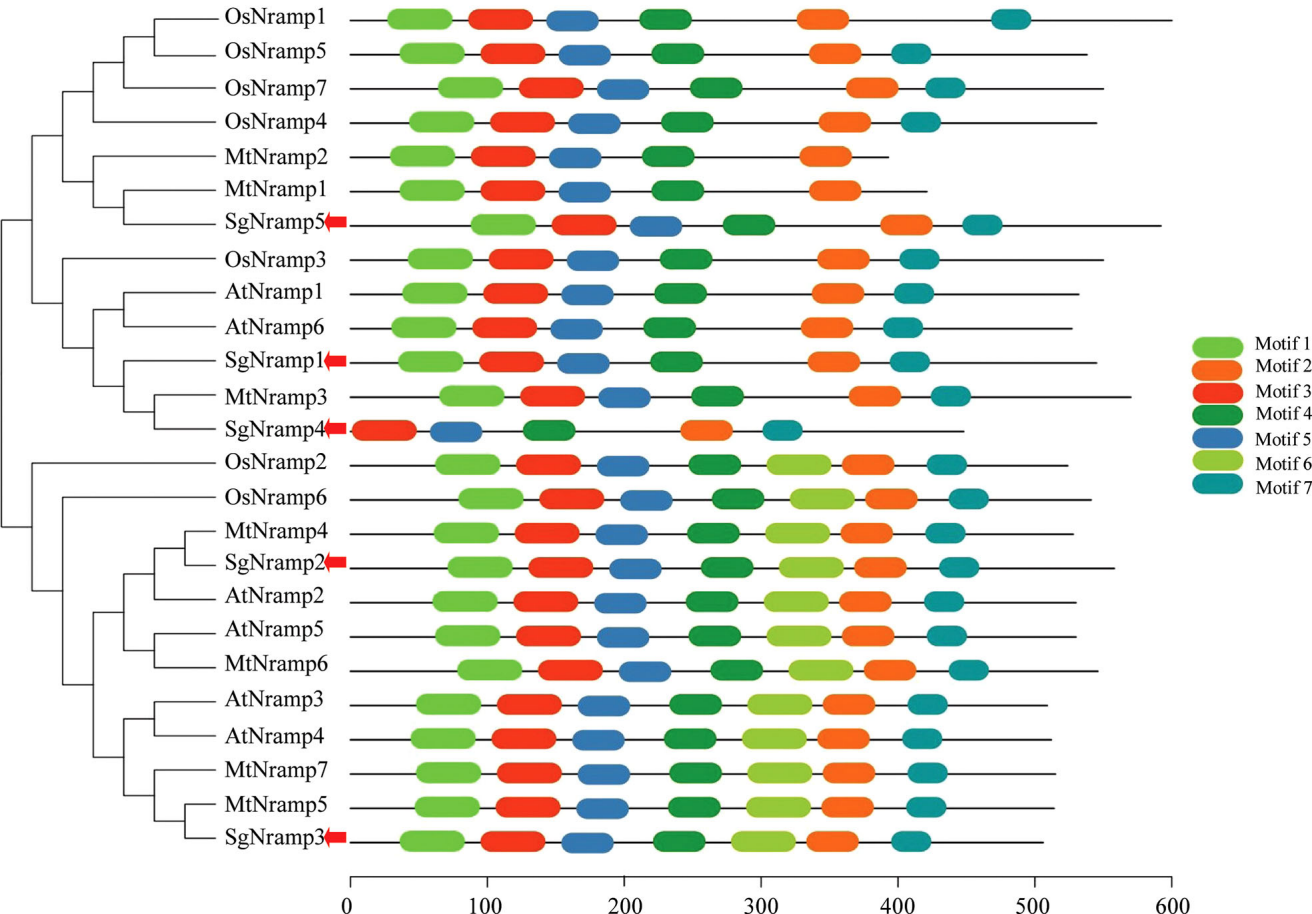
Phylogenetic analysis and conserved motifs of Nramp proteins in stylo and other plants. The first two letters of each Nramp member indicate the plant species. At, *Arabidopsis thaliana*; Os, *Oryza sativa*; Mt, *Medicago truncatula*. The red arrows indicate SgNramps from stylo. The sequences of Nramp proteins were obtained from the previous studies (Tejada-Jiménez et al., 2015; Qin et al., 2017; Mani and Sankaranarayanan, 2018). The phylogenetic tree was constructed by MEGA4. Conserved motifs of Nramp proteins were analyzed by MEME. Each motif is presented by a colored box. The sequences of different motifs of Nramp proteins were summarized in **Supplementary Table S3**.

Phylogenetic analysis was performed using Nramp members from stylo, Arabidopsis, rice, and *M. truncatula*. Results showed that Nramp proteins were divided into two major groups (Groups I and II) (**Figure 2**). Group I included three SgNramps (SgNramp1, 4 and 5), two Nramp proteins from Arabidopsis, five Nramp members from rice, and three Nramps from *M. truncatula*. SgNramp2 and SgNramp3 clustered with four Nramp members from Arabidopsis, two Nramp members from rice, and four Nramps from *M. truncatula* were classified into Group II (**Figure 2**). Interestingly, Nramp members in Group I contained five to six conserved motifs, whereas all Nramp members in Group II contained seven conserved motifs. Among them, SgNramp1 and SgNramp5 included six conserved motifs, whereas SgNramp4 only harbored five conserved motifs. SgNramp2 and SgNramp3 contained seven conserved motifs (**Figure 2** and **Supplementary Table S3**). The conserved motifs included 32–50 amino acids, and putative Nramp domain was predicted in the conserved motifs (**Supplementary Table S3**).

### Expressions of *SgNramps* in different tissues and their responses to metal stresses

**Figure 3:**
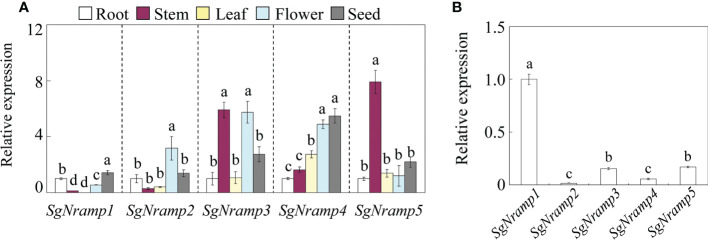
Expression analysis of *SgNramps* in various tissues. **(A)** Expressions of *SgNramps* in root, stem, leaf, flower, and seed. The expression of each gene in the roots was set to one, and then the expression level of each gene in other tissues was normalized to its expression in the roots. **(B)** Expressions of *SgNramps* in stylo root. The expression of *SgNramp1* was set to one, and then the expression level of the other *SgNramps* was normalized to *SgNramp1*. Data are means of three biological replicates with standard error (SE). Different letters represent significant differences among various tissue **(A)** or *SgNramp* genes **(B)** at *P* < 0.05.

The expression patterns of *SgNramps* in tissues of root, stem, leaf, flower, and seed of stylo were measured *via* qRT-PCR. Results showed that *SgNramps* exhibited different expressions in various tissues of stylo (**Figure 3**). Among them, *SgNramp1* mainly expressed in root and seed and exhibited the lowest expression in leaf. *SgNramp2* displayed preferential expressions in flower. The expressions of *SgNramp3* in stem and flower were higher than those of other tissues, whereas *SgNramp4* showed the higher level of expression in flower and seed compared with the other tissues. *SgNramp5* exhibited a higher expression in stem than in the other tissues (**Figure 3**).

**Figure 4:**
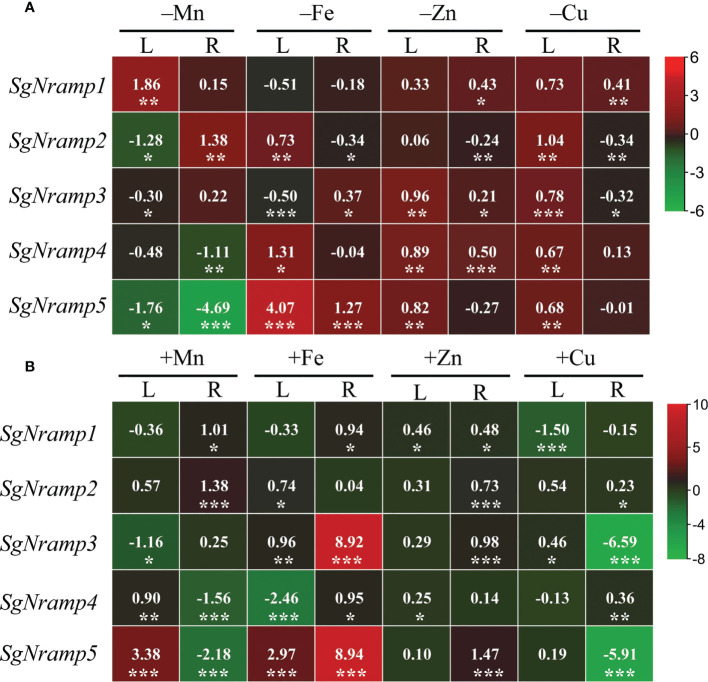
Expression analyses of *SgNramps* in leaf and root of stylo under metal stresses. **(A)** Expressions of *SgNramps* in leaf (L) and root (R) of stylo grown in nutrient solution without MnSO_4_ (–Mn), Fe-Na-EDTA (–Fe), ZnSO_4_ (–Zn), or CuSO_4_ (–Cu) applications for 7 days. **(B)** Expressions of *SgNramps* in leaf (L) and root (R) of stylo grown in nutrient solution supplied with 400 μM MnSO_4_ (+Mn), 800 μM Fe-Na-EDTA (+Fe), 20 μM ZnSO_4_ (+Zn), or 10 μM CuSO_4_ (+Cu) for 7 days. Stylo seedlings grown in full-strength Hoagland solution were set as the control treatment. The data include three biological replicates. The values represented the fold change of each *SgNramp* expression between metal stress and the control (CK), which were calculated as log_2_(metal stress/CK). The positive and negative values represent up- and down-regulation, respectively. Asterisks below the values indicate significant differences between metal stress and CK. *P < 0.05; **0.001 < *P* < 0.01; ***P < 0.001.

To investigate the responses of *SgNramps* to trace metal stresses, including deficient and excess Mn, Fe, Zn, and Cu treatments, the transcription profiles of *SgNramps* were measured in leaf and root. Results showed that the transcript of each *SgNramp* gene was regulated by at least one metal stress in leaf and/or root (**Figure 4**). For example, *SgNramp1* was enhanced, and *SgNramp2*, *SgNramp3* and *SgNramp5* were suppressed by Mn limitation in leaf, whereas *SgNramp2* was enhanced but *SgNramp4* and *SgNramp5* were suppressed under Mn deficient condition in root (**Figure 4A**). In addition, the number of *SgNramps* regulated by Mn stress with more than twofold (the absolute value of log_2_ ≥ 1) was higher than those in Fe, Zn, and Cu stresses. For example, a total of 6, 3, and 1 *SgNramp* genes were regulated by deficiencies of Mn, Fe, and Cu in leaf and/or root, respectively, whereas the transcripts of 6, 4, and 3 *SgNramp* genes were regulated by excess Mn, Fe, and Cu treatments in leaf and/or root, respectively (**Figures 4A, B**). Interestingly, the transcripts of *SgNramp5* were enhanced by more than twofold under limited or excess Fe stress in both leaf and root (**Figure 4**).

### Genotypic differences in *SgNramp1* expression and root Mn concentration

**Figure 5:**
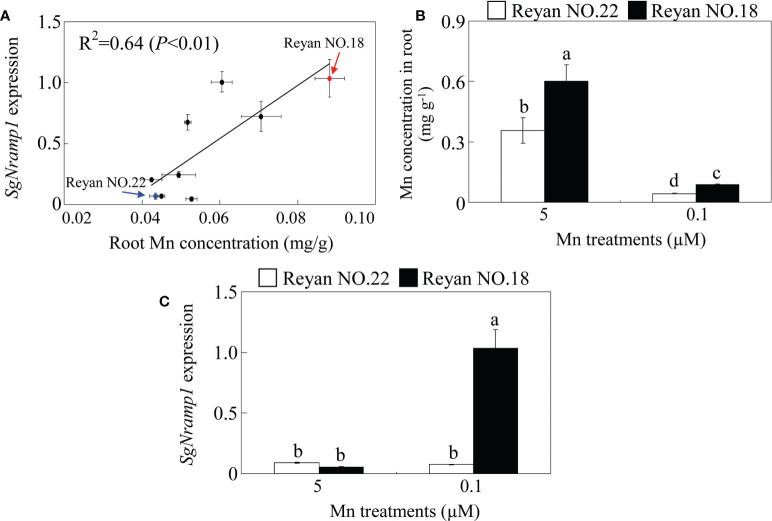
Genotypic variation analysis of *SgNramp1*. **(A)** Analysis of correlation between *SgNramp1* expression and root Mn concentration in various stylo cultivars under 0.1 μM MnSO_4_ treatments. The red and blue arrows indicate Reyan NO.18 and Reyan NO.22 cultivars, respectively. **(B)** Mn concentration in roots of Reyan NO.18 and Reyan NO.22 under 0.1 or 5 μM MnSO_4_ treatments. **(C)** Expression of *SgNramp1* in roots of Reyan NO.18 and Reyan NO.22 under 0.1 or 5 μM MnSO_4_ treatments. Fourteen-day-old stylo seedlings were subjected to 0.1 or 5 μM MnSO_4_ treatments for 7 days that were considered as low Mn and normal Mn treatments, respectively. Stylo roots were used for *SgNramp1* expression analysis and Mn determination. Data are means of three biological replicates with SE. Different letters represent significant differences at *P* < 0.05.

Of the five *SgNramps*, *SgNramp1* displayed high similarity with AtNramp1 that is responsible for Mn uptake in roots of Arabidopsis (**Supplementary Figure S1**). Furthermore, *SgNramp1* displayed the highest expression in stylo roots compared with the other four *SgNramps* (**Figure 3B**). Thus, we further assessed whether *SgNramp1* is responsible for genotypic difference in Mn uptake in various stylo cultivars. Results showed that a close correlation (*R* ^2^ = 0.64, *P* < 0.01) was found between *SgNramp1* expression and root Mn concentration in nine stylo cultivars under 0.1 μM MnSO_4_ condition (low Mn treatment) (**Figure 5A**).

Among the tested nine stylo cultivars, two cultivars, Reyan NO.18 and Reyan NO.22, exhibited significant genotypic variation in *SgNramp1* expression and root Mn concentration. Furthermore, Mn concentration in root of Reyan NO.18 was significantly higher than that in Reyan NO.22 under both 0.1 and 5 μM MnSO_4_ treatments, which were considered as low Mn or normal Mn treatments, respectively (**Figure 5B**). In addition, compared with normal Mn treatment, the expressions of *SgNramp1* were significantly increased by low Mn in Reyan NO.18 but not in Reyan NO.22 (**Figure 5C**). Therefore, SgNramp1 is likely to be involved in Mn uptake in stylo and was then selected for further analysis.

### Subcellular localization of SgNramp1

**Figure 6:**
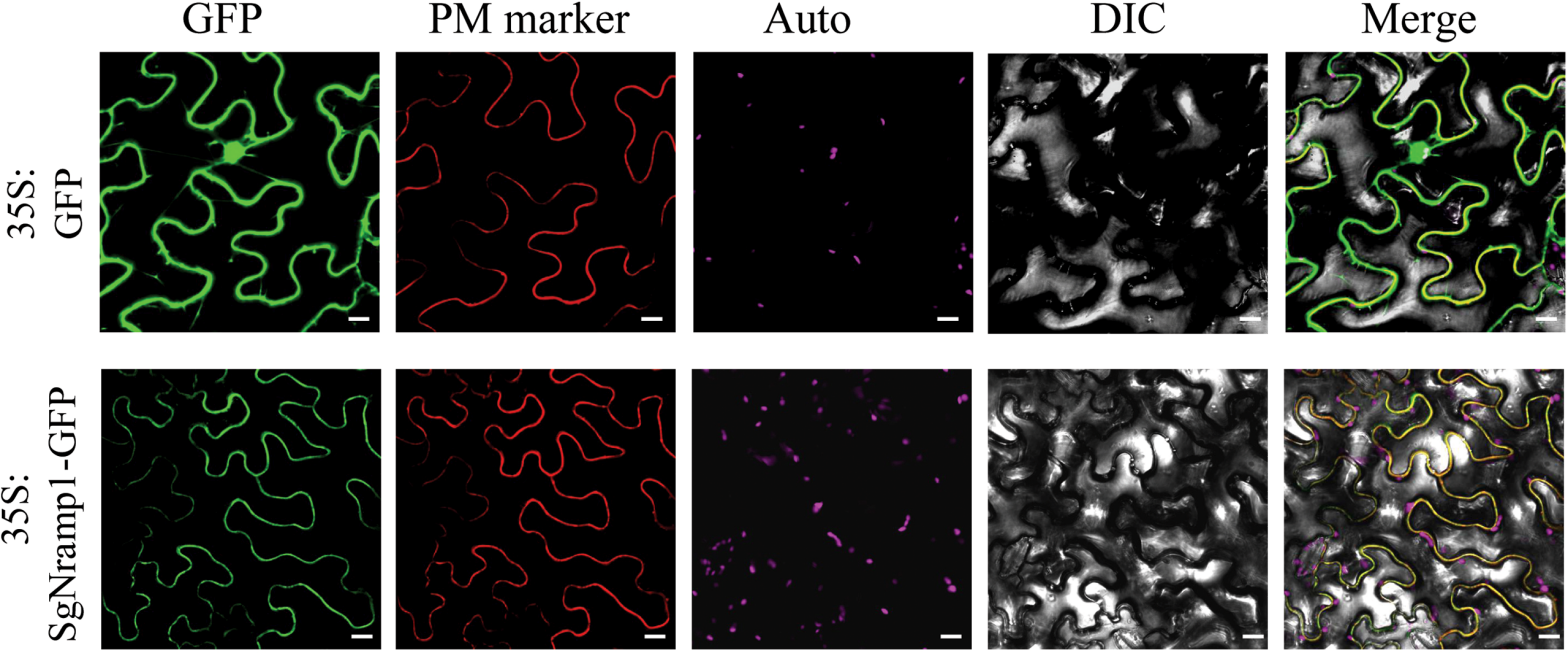
Subcellular localization of SgNramp1. The *35:SgNramp1-GFP* construct and *35:GFP* empty vector were transiently expressed in tobacco epidermis cells. SgNramp1 was co-expressed with the plasma membrane (PM) marker, which was fused with red fluorescence protein (mKATE). Signals of GFP fusion protein, PM marker, chlorophyll autofluorescence (Auto), bright-field images (DIC), and the merged images (Merge) were shown from left to right. Scale bar is 20 μm.

*SgNramp1* was cloned and fused to the N-terminus of GFP (*35S:SgNramp1-GFP*). Then, subcellular localization analysis was performed in tobacco epidermis cells through transiently expressed *35S:SgNramp1-GFP* and empty vector. Results showed that the fluorescence of GFP alone in epidermis cells was found in the cytoplasm, nucleus, and PM, whereas GPF fluorescence of SgNramp1 was found to be co-localized with that of the PM marker in the epidermis cells (**Figure 6**), suggesting that SgNramp1 is a PM–localized protein.

### Heterologous expression of *SgNramp1* in yeast cells

**Figure 7:**
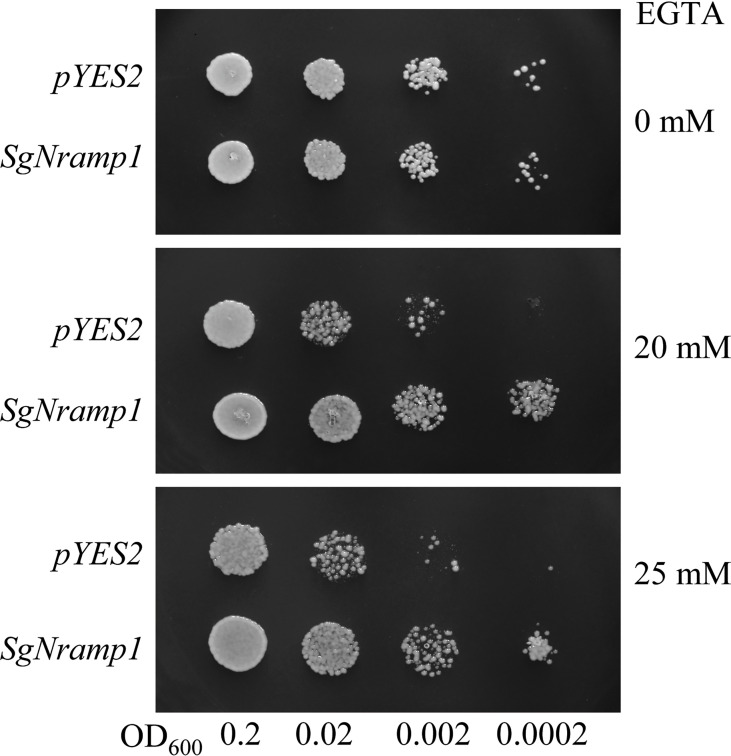
Manganese transport analysis of SgNramp1 in yeast. The yeast mutant Δ*smf1* expressing either the *pYES2* empty vector or *SgNramp1* was spotted on SC-U/Gal solid medium containing 0, 20, or 25 mM Mn-chelator EGTA. The photo shows the growth of yeast cells at 30°C for 2 days.

Subsequently, the coding sequence of *SgNramp1* was cloned into the *pYES2* vector and transformed into various yeast strains. As shown in **Figure 7**, no differences in the growth were observed in the Mn uptake-defective yeast mutants Δ*smf1* expressing either the *pYES2* empty vector or *SgNramp1* gene in the control medium without EGTA supplement. Under Mn limited condition by added with 20 or 25 mM EGTA, *SgNramp1* expression rescued the growth of the yeast strain Δ*smf1* compared with the empty vector control, although the growth of both strains was inhibited by low Mn stress (**Figure 7**). No significant differences in growth performance were observed between the Fe uptake-defective yeast mutants Δ*fet3fet4* transformed with either the *pYES2* empty vector or *SgNramp1* in the medium with 0, 20, or 40 μМ Fe supplements (**Supplementary Figure S2A**). In addition, the Cd-sensitive yeast strains Δ*ycf1* expressing either the *pYES2* empty vector or *SgNramp1* exhibited the same sensitivity to Cd stress in the medium added with 20 or 60 μМ Cd (**Supplementary Figure S2B**). These results suggest that *SgNramp1* can transport Mn but not Fe and Cd.

**Figure 8:**
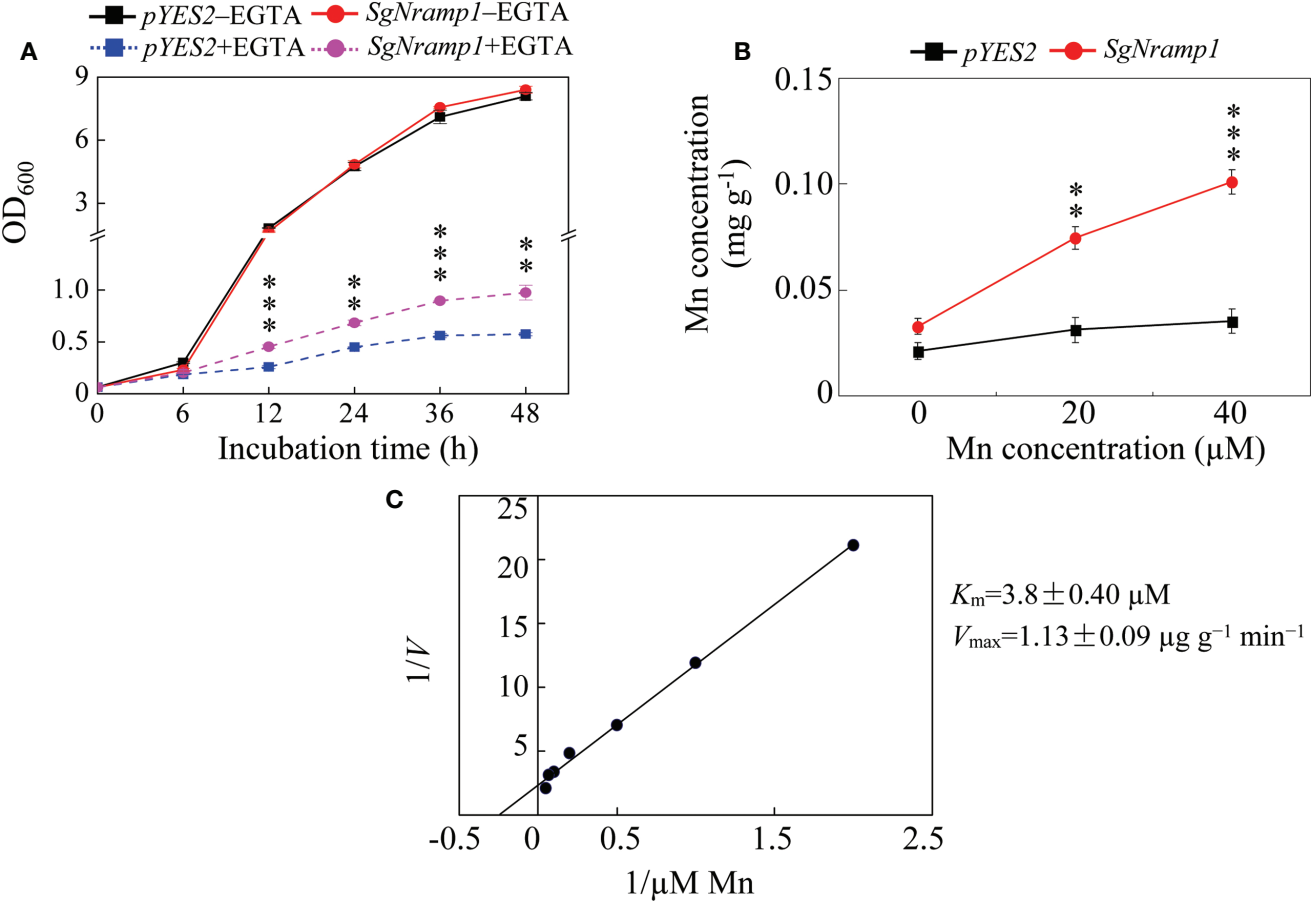
Growth and Mn uptake in yeast mutant Δ*smf1* expressing *SgNramp1*. **(A)** The yeast mutant Δ*smf1* expressing either the *pYES2* empty vector or *SgNramp1* was grown in SC-U/Gal liquid medium with or without 20 mM EGTA for 0h to 48h. **(B)** Mn concentrations in the yeast cells grown in SC-U/Gal liquid medium containing 0, 20, or 40 μМ MnSO_4_ for 24h. **(C)** The kinetics of Mn uptake in yeast cells. The kinetics parameters are shown next to the figure. Data are means of three biological replicates with SE. Asterisks indicate significant differences between the *pYES2* empty vector and *SgNramp1*. **0.001 < *P* < 0.01; ***P < 0.001.

To further investigate the differences in yeast growth and Mn uptake, the yeast strains Δ*smf1* expressing the *pYES2* empty vector or *SgNramp1* were grown in a liquid SC-U/Gal medium added with or without 20 mМ EGTA. The growth curve showed the similar growth status of the two strains under without EGTA condition (**Figure 8A**). In the presence of 20 mМ EGTA, the growth of yeast strain expressing *SgNramp1* was better than the yeast strain transformed with the empty vector control. The OD_600_ values of yeast cells expressing *SgNramp1* were 52.3 and 69.8% higher than that of the yeast strain transformed with the empty vector control at 24 h and 48 h of incubation, respectively (**Figure 8A**). Furthermore, Mn concentration in the yeast cells expressing *SgNramp1* was 1.4- and 1.9-fold higher than those in the yeast cells transformed with the empty vector control supplemented with 20 and 40 μМ MnSO_4_, respectively (**Figure 8B**). In addition, kinetic analysis of Mn uptake in yeast showed that the *K*_m_ value was 3.8 ± 0.40 μM and the *V*_max_ value was 1.13 ± 0.09 μg g^−1^ min^−1^ (**Figure 8C**). Thus, SgNramp1 is involved in Mn uptake in yeast cells.

## Discussion

Numerous studies demonstrated that Nramp proteins participate in the uptake, translocation, and distribution of metal ions, regulating metal homeostasis in plants (Sasaki et al., 2012; Li et al., 2021c). Although Nramp homologues have been reported in plants, Nramp members and their functional roles in the important tropical legume, stylo, have not been elucidated so far. In this study, we identified five *SgNramp* genes in stylo. All of them possessed the conserved Nramp domain (PF01566) and carried the unique amino acid residues of Nramp proteins (**Figure 1**), which have been widely identified in AtNramps from Arabidopsis, OsNramps from rice, GmNramps from soybean, PvNramps from bean (*Phaseolus vulgaris*), and CsNramps from tea (C*amellia sinensis*) (Thomine et al., 2000; Yamaji et al., 2013; Qin et al., 2017; Ishida et al., 2018; Li et al., 2021b). Furthermore, Nramp proteins have been reported to include 10–12 TMDs. For example, there are 12 TMDs in AtNramp1/2, 10 TMDs in OsNramp5, 11 TMDs in HvNramp5, and 12 TMDs in FeNramp5 (Curie et al., 2000; Sasaki et al., 2012; Wu et al., 2016; Yokosho et al., 2021). Consistently, all of the SgNramp proteins contained 12 conserved TMDs, except SgNramp4, which harbored 10 TMDs (**Figure 1** and **Supplementary Table S2**). These conserved structures are essential for the functions of Nramp proteins.

Phylogenetic analysis showed that SgNramp1 and SgNramp4, together with AtNramp1 and AtNramp6 from Arabidopsis, MtNramp3 from *M. truncatula*, and OsNramp3 from rice were grouped into the same cluster in Group I (**Figure 2**). AtNramp1 is a PM-localized transporter that participates in Mn uptake in Arabidopsis (Cailliatte et al., 2010). AtNramp6 is able to transport Fe and regulates lateral root growth in Arabidopsis under low Fe stress (Cailliatte et al., 2009; Li et al., 2019). Furthermore, AtNramp6 is suggested to cooperate with AtNramp1 in controlling Mn homeostasis in Arabidopsis (Li et al., 2022). OsNramp3 is characterized to be an important node-based transporter essential for preferential Mn distribution, mediating Mn remobilization between young leaves and old tissues of rice in response to variable Mn levels (Yamaji et al., 2013; Yang et al., 2013). As the closest homologue of SgNramp5 in Group I, MtNramp1 is implicated in Fe transport in the yeast cells, and it participates in apoplastic Fe uptake by rhizobia-infected cells in nodules of *M. truncatula* (Tejada-Jiménez et al., 2015). In Group II, SgNramp2 exhibited high similarity to AtNramp2, which is localized to the trans-Golgi network and is proposed to involve in Mn transport in Golgi, regulating root growth of Arabidopsis under low Mn stress (Gao et al., 2018). SgNramp3 was clustered with AtNramp3/4 and MtNramp5/7 in the same subgroup (**Figure 2**). In addition to participate in vacuolar Fe mobilization during seed germination, AtNramp3 and AtNramp4 are demonstrated to be responsible for intracellular Mn homeostasis in Arabidopsis (Thomine et al., 2003; Lanquar et al., 2005; Lanquar et al., 2010). Therefore, stylo SgNramps might possess the potential role in regulating metal homeostasis in stylo.

Nramp proteins have been characterized to function in the regulation of metal uptake and homeostasis, and thus they may exhibit preferential expressions in various plant tissues. For example, a set of *Nramp* homologues are observed to be mainly expressed in roots, such as *AtNramp1* from Arabidopsis (Cailliatte et al., 2010), *OsNramp4/5* from rice (Xia et al., 2010; Sasaki et al., 2012), *MtNramp1* from *M. truncatula* (Tejada-Jiménez et al., 2015), *HvNramp5* from barley (Wu et al., 2016), *TtNramp6* from wheat (*Triticum turgidum*) (Wang et al., 2019a), *AhNramp1* from peanut (*Arachis hypogaea*) (Wang et al., 2019b), *FeNramp5* from buckwheat (Yokosho et al., 2021), and *NtNramp1* from tobacco (Liu et al., 2022). The root-specific expression of *Nramps* is beneficial for metal uptake. Similarly, variations in the abundances of *SgNramps* were observed in different tissues of stylo, such as *SgNramp1* mainly expressed in root and seed (**Figure 3**), suggesting the key role of *SgNramp* members in regulating metal homeostasis in specific tissues.

In addition, *SgNramp* genes exhibited diverse responses to Mn, Fe, Zn, and Cu stresses as well as heavy metal toxicity in stylo (**Figure 4** and **Supplementary Figure S3**). In Arabidopsis, the transcripts of *AtNramp1* in roots are enhanced by both low Fe and Mn levels, whereas both *AtNramp3* and *AtNramp4* abundances are increased in roots and shoots by Fe starvation but not Mn deficiency (Curie et al., 2000; Thomine et al., 2000; Cailliatte et al., 2010). In rice, the expression of *OsNramp5* gene is up-regulated in roots by Zn and Fe deficiencies but not low Mn and Cu conditions (Xia et al., 2010; Sasaki et al., 2012). On the other hand, although some of the *Nramp* homologues are not response to metal stresses, they are able to transport metal ions. For example, *AtNramp2* and *AtNramp6* genes are not regulated by deficiencies of Mn, Fe, and Zn, but *AtNramp2* is found to be involved in remobilization of Mn in Golgi for Arabidopsis root growth (Gao et al., 2018), and *AtNramp6* is shown to participate in regulation of Fe and Mn homeostasis (Li et al., 2019; Li et al., 2022). Although *OsNramp3* is unaffected by deficiencies of Zn, Fe, Mn, and Cu at transcriptional level, its encoding protein is rapidly degraded after high Mn exposure, regulating Mn distribution in young leaves and old tissues (Yamaji et al., 2013). Thus, changes in the abundances of *SgNramp* genes might potentially contribute to regulate metal homeostasis in various tissues of stylo for dealing with the fluctuated metal levels.

In this study, we found that the transcript levels of *SgNramp1* are responsible for the genotypic differences in root Mn levels in stylo, as reflected by a close correlation between *SgNramp1* expression and Mn concentration in various stylo cultivars (**Figure 5**). Furthermore, a higher level of Mn was observed in the stylo cultivar Reyan NO.18 compared with Reyan NO.22, which was correlated with higher *SgNramp1* expression in Reyan NO.18 under low Mn condition (**Figure 5**). Similarly, differences in Mn efficiency have been observed among barley genotypes, which has been attributed to differences in Mn uptake capability; by analyzing two barley genotypes contrasting in Mn acquisition efficiency and gene expression, the role in Mn uptake has been suggested for HvIRT1, which exhibits higher expression in a Mn-efficient barley genotype than a Mn-inefficient genotype and has the ability to restore Mn uptake of Δ*smf1* mutant (Pedas et al., 2005, Pedas et al., 2008).

The metal transport activity of SgNramp1, which was localized to the PM, was further analyzed in yeast cells. Heterologous expression of *SgNramp1* in yeast mutants showed that *SgNramp1* can restore the growth of the yeast strain Δ*smf1* through increasing Mn uptake (**Figures 7** and 8). It has been demonstrated that a homologue of *SgNramp1*, *AtNramp1*, encoding a PM-localized transporter, can restore the growth of yeast mutant Δ*smf1* under Mn limitation (Curie et al., 2000; Thomine et al., 2000). Furthermore, loss function of *AtNramp1* disrupts the growth and Mn concentration in Arabidopsis under low Mn condition, demonstrating that AtNramp1 is the transporter responsible for Mn uptake in Arabidopsis (Cailliatte et al., 2010). In addition, broad transport substrates of Nramp homologues were observed in other plants, such *M. truncatula*, rice, barley, and buckwheat (Ishimaru et al., 2012; Tejada-Jiménez et al., 2015; Wu et al., 2016; Yokosho et al., 2021). For example, OsNramp5 in rice transports Mn, Cd, and Fe (Ishimaru et al., 2012), whereas HvNramp5 in barley and FeNramp5 in buckwheat only transport Mn and Cd but not Fe (Wu et al., 2016; Yokosho et al., 2021). Thus, it is reasonable to assume that *SgNramp1* might contribute to Mn acquisition in stylo. However, the function of *SgNramp1* may be differed in various stylo cultivars. Further research work is needed, such as generation of transgenic stylo plants overexpressing or suppressing *SgNramp1*, to verify its exact function conducted in both nutrient solutions and soil conditions with different Mn status and availability.

In summary, we identified five *SgNramp* genes in stylo. Changes observed in the abundances of *SgNramps* suggest the involvement of these genes in regulating metal homeostasis in tissues of different plant parts of stylo during metal stress conditions. Moreover, SgNramp1 is a PM-localized transporter and it is responsible for Mn uptake. Increases of *SgNramp1* expression may contribute to enhance the acquisition of Mn in stylo. Therefore, *SgNramp1* might be a good candidate for breeding future stylo cultivars with efficient Mn uptake through gene-editing technology.

## Data availability statement

The datasets presented in this study can be found in online repositories. The names of the repository/repositories and accession number(s) can be found in the article/**Supplementary Material**.

## Author contributions

ZC designed the research. XZ, RH, LW and GW performed the experiments and analyzed the data. YM and GL prepared the plant material for this work. XZ, RH and ZC wrote the manuscript. IR, GL and ZC discussed and revised the manuscript. All authors contributed to the article and approved the submitted version.

## Funding

This work was funded by the National Natural Science Foundation of China (32271756, 31861143013), the National Natural Science Foundation of Hainan (321RC645), and the China Agriculture Research System of MOF and MARA (CARS-34).

## Acknowledgments

The authors appreciate Yufen Xing for technical help.

## Conflict of interest

The authors declare that the research was conducted in the absence of any commercial or financial relationships that could be construed as a potential conflict of interest.

## Publisher’s note

All claims expressed in this article are solely those of the authors and do not necessarily represent those of their affiliated organizations, or those of the publisher, the editors and the reviewers. Any product that may be evaluated in this article, or claim that may be made by its manufacturer, is not guaranteed or endorsed by the publisher.

## Supplementary material

Supplementary Figure 1 Homology identity analysis of SgNramp proteins. **(A)** Homology identity among SgNramp proteins. **(B)** Homology identity of SgNramps with AtNramp1 (accession no. At1g80830) from Arabidopsis. **(C)** Homology identity of SgNramps with MtNramp1 (accession no. Medtr3g088460) from *M. truncatula*. **(D)** Homology identity of SgNramps with GmDMT1 (accession no. AY169405) from soybean. The homology identity (%) was analyzed by Clustal.

Supplementary Figure 2 Transport activity of SgNramp1 in yeast mutants Δ*fet3fet4* and Δ*ycf1*. **(A)** The yeast mutant Δ*fet3fet4* defective in Fe uptake expressing either the *pYES2* empty vector or *SgNramp1* was grown in SC-U/Gal medium containing 0, 20, or 40 μМ Fe. **(B)** The yeast mutant Δ*ycf1* sensitive in Cd stress expressing either the *pYES2* empty vector or *SgNramp1* was grown in SC-U/Gal medium containing 0, 20, or 60 μМ Cd. The photo shows the growth of yeast cells at 30°C for 2 days.

Supplementary Figure 3 Expression of *SgNramps* in roots of stylo under heavy metal treatments. Fourteen-day-old stylo seedlings were separately subjected to 0 (CK), 100 μM AlCl_3_, 40 μM CdCl_2_, or 20 μM LaCl_3_ treatments for 2 days. Roots were harvested for gene expression analysis. Data are means of three biological replicates with standard error (SE). Asterisks indicate significant differences between control and treatments. *0.01 < *P* < 0.05; **0.001 < *P* < 0.01; ****P* < 0.001.
