# Supplementary material for: SgNramp1, a plasma membrane-localized transporter, involves in manganese uptake in Stylosanthes guianensis
Source: Front Plant Sci. 2022 Oct 6;13:1027551. doi: 10.3389/fpls.2022.1027551 (PMC9583531; doi:10.3389/fpls.2022.1027551)
Supplement: Supplementary Figure 1 — Homology identity analysis of SgNramp proteins. (A) Homology identity among SgNramp proteins. (B) Homology identity of SgNramps with AtNramp1 (accession no. At1g80830) from Arabidopsis. (C) Homology identity of SgNramps with MtNramp1 (accession no. Medtr3g088460) from M. truncatula. (D) Homology identity of SgNramps with GmDMT1 (accession no. AY169405) from soybean. The homology identity (%) was analyzed by Clustal. [file DataSheet_1.pdf]

Supplementary Figure S1

A

Proteins	SgNramp1	SgNramp2	SgNramp3	SgNramp4	SgNramp5
SgNramp1	100.0%	32.7%	35.0%	81.2%	55.9%
SgNramp2	32.7%	100.0%	73.7%	33.3%	32.1%
SgNramp3	35.0%	73.7%	100.0%	31.8%	34.3%
SgNramp4	81.2%	33.3%	31.8%	100.0%	60.0%
SgNramp5	55.9%	32.1%	34.2%	60.0%	100.0%

B

Proteins	AtNramp1
SgNramp1	75.9%
SgNramp2	32.1%
SgNramp3	33.9%
SgNramp4	77.0%
SgNramp5	56.2%

C

Proteins	MtNramp1
SgNramp1	57.2%
SgNramp2	35.9%
SgNramp3	36.3%
SgNramp4	48.2%
SgNramp5	76.0%

D

Proteins	GmDMT1
SgNramp1	34.1%
SgNramp2	72.9%
SgNramp3	81.8%
SgNramp4	31.5%
SgNramp5	34.1%
