# Supplementary figures and images for: SgNramp1, a plasma membrane-localized transporter, involves in manganese uptake in Stylosanthes guianensis
Source: Front Plant Sci. 2022 Oct 6;13:1027551. doi: 10.3389/fpls.2022.1027551 (PMC9583531; doi:10.3389/fpls.2022.1027551)

## Supplementary Figure S2

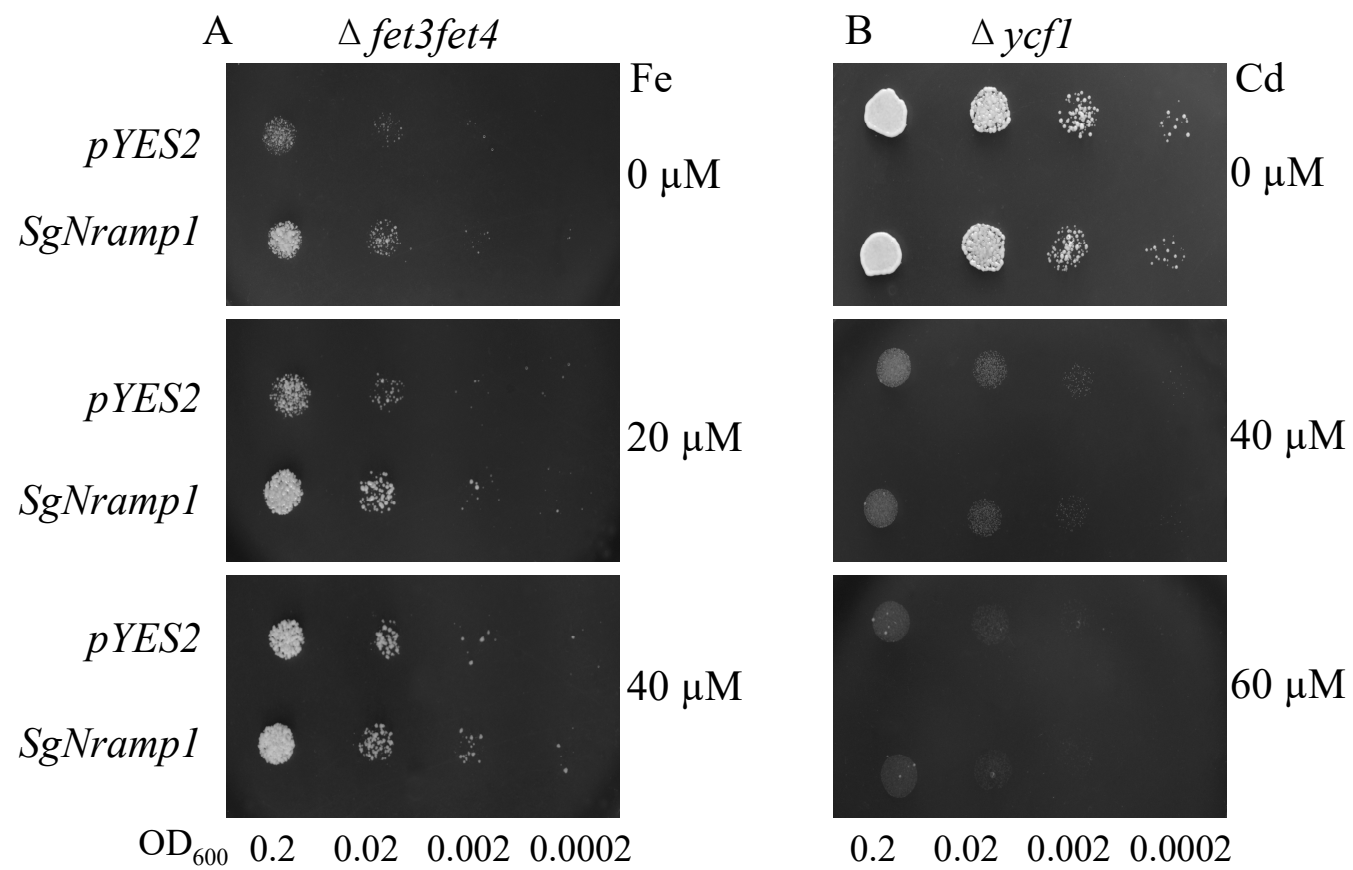

Supplement: Supplementary Figure 2 — Transport activity of SgNramp1 in yeast mutants Δfet3fet4 and Δycf1. (A) The yeast mutant Δfet3fet4 defective in Fe uptake expressing either the pYES2 empty vector or SgNramp1 was grown in SC-U/Gal medium containing 0, 20, or 40 μМ Fe. (B) The yeast mutant Δycf1 sensitive in Cd stress expressing either the pYES2 empty vector or SgNramp1 was grown in SC-U/Gal medium containing 0, 20, or 60 μМ Cd. The photo shows the growth of yeast cells at 30°C for 2 days. [file DataSheet_2.pdf]

Supplementary Figure S3

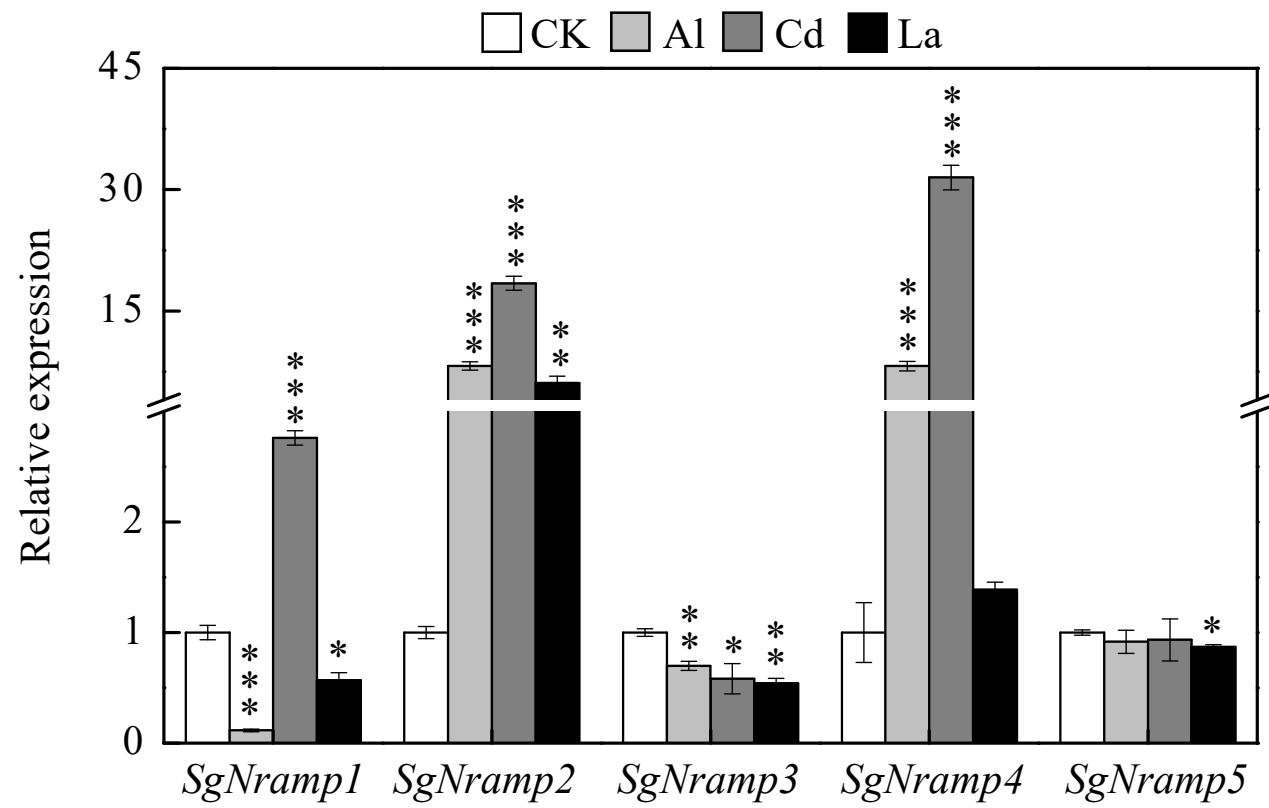

Supplement: Supplementary Figure 3 — Expression of SgNramps in roots of stylo under heavy metal treatments. Fourteen-day-old stylo seedlings were separately subjected to 0 (CK), 100 μM AlCl3, 40 μM CdCl2, or 20 μM LaCl3 treatments for 2 days. Roots were harvested for gene expression analysis. Data are means of three biological replicates with standard error (SE). Asterisks indicate significant differences between control and treatments. *0.01 < P < 0.05; **0.001 < P < 0.01; ***P < 0.001. [file DataSheet_3.pdf]
