# Supplementary material for: SgNramp1, a plasma membrane-localized transporter, involves in manganese uptake in Stylosanthes guianensis
Source: Front Plant Sci. 2022 Oct 6;13:1027551. doi: 10.3389/fpls.2022.1027551 (PMC9583531; doi:10.3389/fpls.2022.1027551)
Supplement: Supplementary file 4 [file DataSheet_4.doc]

**Table S1 Primers used in study.**

| **primer name** | **Forward primer (F, 5’-3’)** | **Reverse primer (R, 5’-3’)** |
| --- | --- | --- |
| *SgNramp1-RT* | GCGCTTCCGTTCGCTTTGATT | CTTCCGGAACACGAGGTATGCAA |
| *SgNramp2-RT* | TTGGGCGAGGCTAGTTCTCTGG | CGACACCGACCGCTTGATGAAT |
| *SgNramp3-RT* | CCGTGTTCGCTAAGGGCTTT | CTGCTGCTAACAATCCAATGC |
| *SgNramp4-RT* | TGCTCTTATGGTGGCCTTCCTCA | TCCCTGCATGACATACTGCCCTG |
| *SgNramp5-RT* | ATCACCGATACGAGCTGCTGTG | TGTTTCCCTGTGCTTACGCCAA |
| *SgNramp1-GFP* | CAGTGGTCTCACAACATGGCTCCAGAGCAGCCACA | CAGTGGTCTCATACAAATCTCTGCTGGACTAATAA |
| *SgNramp1-pYES2* | GGATCCAAAAAAATGTCTGCTCCAGAGCAGCCACA | TCTAGATCAAATCTCTGCTGGACTAA |
| *SgEF1a-RT* | CACTTCAGGACGTGTACAAGATC | CTTGGAGAGCTTCATGGTGCA |

**Table S2** General information for the *SgNramps* in *S.* *guianensis*.

| **Gene Name** | **ORF Length (bp)** | **AA** | **MW (kDa)** | **p*I*** | **TMD** |
| --- | --- | --- | --- | --- | --- |
| *SgNramp1* | 1635 | 544 | 59.5 | 8.2 | 12 |
| *SgNramp2* | 1674 | 557 | 61.0 | 5.2 | 12 |
| *SgNramp3* | 1518 | 505 | 55.2 | 5.2 | 12 |
| *SgNramp4* | 1344 | 447 | 48.3 | 9.1 | 10 |
| *SgNramp5* | 1779 | 591 | 64.5 | 9.2 | 12 |

Note, ORF, Open reading frame. AA, Length of the amino acid sequence. MW, Molecular weight. p*I*, Isoelectric point. TMD, Transmembrane domain.

**Table S3 Sequences of different motifs of Nramp proteins.**

| **Motif** | **Length** | **Protein Sequences** | **Pfam Domain** |
| --- | --- | --- | --- |
| 1 | 50 | PPFSWKKLWLFTGPGFLMSIAFLDPGNLETDLQAGAIAGYELLWLJLWAT | Nramp |
| 2 | 41 | GQSSTITGTYAGQFIMQGFLBLRLKKWJRNLITRSLAIVPT | Nramp |
| 3 | 50 | AARLGVATGRHLAELCREEYPKWARYVLWLMAELALIAADIPEVIGTAIA | Nramp |
| 4 | 41 | GAIGQAVGLVGALIMPHNLFLHSALVLSRKIPRSKKGRVZE | Nramp |
| 5 | 41 | PLWAGVLJTGLSTFJFLFLZNYGVRKLEALIAVLIATMALC | Nramp |
| 6 | 50 | FMINJFVTTVFAKGFYGTEQABSIGLVNAGQYLQEKYGGGVFPILYIWGI | Nramp |
| 7 | 32 | NVJQSFZJPFALIPLLKLVSKETKMGSFKIGP | Nramp |

Note, conserved motifs of Nramp proteins were analyzed by MEME and Pfam.
